# Supplementary material for: The effects of microbiome-targeted therapy on cognitive impairment and postoperative cognitive dysfunction—A systematic review
Source: PLoS One. 2023 Feb 7;18(2):e0281049. doi: 10.1371/journal.pone.0281049 (PMC9904456; doi:10.1371/journal.pone.0281049)
Supplement: S2 File — (PDF) [file pone.0281049.s002.pdf]

Search Strategies Appendix

Database	Search Strategy
PubMed	(microbiome OR "Microbiota"[Mesh] OR "Gastrointestinal Microbiome"[Mesh] OR probiotic OR probiotics OR "Probiotics"[Mesh]) AND ("cognitive dysfunction"[mesh] OR “cognitive dysfunction” Or "Delirium"[mesh] OR delirium OR "cognitive impairment" OR confusion OR “mental deterioration” OR “Cognition Disorders”[Mesh] OR “Anesthesia/adverse effects”[Mesh] OR “Anesthetics/adverse effects”[Mesh] OR “Postoperative complications/therapy”[Mesh] OR “Postoperative complications/prevention and control”[Mesh] OR “Dysbiosis/therapy”[Mesh] OR “Inflammation/drug therapy”[Mesh] OR “Inflammation/complications”[Mesh]) AND ("Aged"[Mesh] OR aged OR elderly OR geriatric)
Web of Science	(microbiome OR "Microbiota" OR "Gastrointestinal Microbiome" OR probiotic OR probiotics) AND (((((((“cognitive dysfunction” OR delirium OR "cognitive impairment" OR confusion OR “mental deterioration” OR “Cognition Disorders”) OR (Anesthesia AND “adverse effects”) OR (Anesthetics AND “adverse effects”) OR (“Postoperative complications” AND therapy) OR (“Postoperative complications” AND prevention) OR (Dysbiosis AND therapy) OR (Inflammation AND “drug therapy”) OR (Inflammation AND complications))))))) AND (Aged OR elderly OR geriatric)
Embase	('microbiome'/exp OR microbiome OR 'microflora'/exp OR microflora OR 'intestine flora'/exp OR 'intestine flora' OR 'probiotic agent'/exp OR 'probiotic agent') AND ('delirium'/exp OR delirium OR 'cognitive defect'/exp OR 'cognitive defect' OR 'confusion'/exp OR confusion OR 'mental deterioration'/exp OR 'mental deterioration' OR 'anesthesia'/exp/dd_ae OR 'anesthetic agent'/exp/dd_ae OR 'postoperative complication'/exp/dm_pc OR 'dysbiosis'/exp/dm_th OR 'inflammation'/exp/dm_co,dm_dt) AND ('aged'/exp OR 'elderly'/exp OR 'geriatrics'/exp)
Cochrane Library	(Microbiome “cognitive defect” elderly OR Microbiome anesthesia adverse effects elderly OR Microbiome Dysbiosis elderly OR Microbiome inflammation elderly OR Microbiota “postoperative complications” elderly OR Microbiota inflammation elderly OR Probiotic delirium elderly OR Probiotic confusion elderly OR Probiotic “postoperative complications” elderly OR Probiotic Dysbiosis elderly OR Probiotic inflammation elderly)
