# Supplementary material for: The effects of microbiome-targeted therapy on cognitive impairment and postoperative cognitive dysfunction—A systematic review
Source: PLoS One. 2023 Feb 7;18(2):e0281049. doi: 10.1371/journal.pone.0281049 (PMC9904456; doi:10.1371/journal.pone.0281049)
Supplement: S3 File — (PDF) [file pone.0281049.s003.pdf]

PROTOCOL SYNOPSIS

Review title	The Effects of Microbiome-Targeted Therapy on Cognitive Impairment and Postoperative Cognitive Dysfunction - A Systematic Review
Princpal investigator<br>Organizational afflication<br>Review team members<br>and their afflication<br>Fundung Sources<br>Conflicts of interest	Saiko Sugita, M.D., Ph.D.<br>University of California, San Francisco<br>Sakura Kinjo, M.D., University of Calicornia, San Francisco<br>Peggy Tahir, MLIS, MA., University of California, San Francisco<br>None<br>None
Review question(s)	Could microbiome-targeted therapy (MTT) including supplementation with probiotics and prebiotics be a preventive strategy for cognitive deterioration in rodent model? Especially for the cognitive decline accompanied by aging, postoperative delirium (POD), postoperative cognitive dysfunction (POCD), Alzheimer's disease (AD), and dementia.
Context and Rationte	POCD has become a significant problem because of increasing surgical procedures among aging patients. Prolonged POCD can lead to a higher risk of permanent cognitive impairment or dementia. Due to the increase of lifespan, identifying preventive strategies for POCD has become a important issue. The concept of the gut-brain axis, the bidirectional communication between gut microbiota in the gastrointestinal tract and brain has recently been confirmed by a growing number of studies. Accordingly, the link between gut microbiota and POCD has been getting attention.<br>Our final purpose of this review is assessing if microbiome-targeted therapies could be a preventive strategy for PND that occurs in elderly patientsat a high probability. Aging, and pre-exsisting cognitive impairment such as AD and dementia are consistantly reported risk factors for POD and POCD. The pathological cause of POCD is believed to be neuroinflammation, which is also a hallmark of Alzheimer’s disease. A Senescence Accelerated Mouse-Prone 8 (SAMP8) mouse displays a phenotype of accelerated aging.<br>Therefore we will analyze studies in rodent model with cognitive disorder including SAMP8, AD, and dementia models.
Searches	We will conduct the search according to he guidelines of the Preferred Reporting Items for Systematic Reviews and Meta-Analyses (PRISMA) checklist. Two authors (S.S., P.T.) will separately search for publications using PubMed, EMBASE, Cochrane, and Web of Science for relevant literature (from their inception to October 2021) using the following terms : microbiome, microbiota, gastrointestinal microbiome, probiotic, probiotics, cognitive dysfunction, delirium, cognitive impairment, confusion, mental deterioration, cognition disorders, anesthesia/adverse effects, postoperative complications/therapy, postoperative complications/prevention and control, dysbiosis/therapy, inflammation/drug therapy, inflammation/complications, aged, elderly and geriatric.<br>We will exclude publications not written in English or no available abstract or full text online.
Human disease modelled	Postoperative delirium, Postoperative cognitive dysfunction, AD, Dementia
Animals/ Population(P)	Inclusion criteria: 1) Rodent models with MTT in perioperative settings.<br>2) Rodent models with cognitive deterioration including AD, dementia, or SAMP8.<br>Exclusion criteria: 1) Animal models other than rodent.<br>2) Rodent models with cognitive decline associated with disease other than the above, such as hepatic encephalopathy, HIV-associated cognitive decline.
Interventions(I)	Inclusion criteria: microbiome-targeted therapy such as su supplementation using probiotics and/or prebiotics, or fecal microbiome transplantation.<br>Exclusion criteria: Probiotic or prebiotic use without specification of their names.
Comparator(s)/control(C)	Inclusion criteria: Randomly assigned placebo, sham or germ-free rodents<br>Exclusion criteria: 1) Not randomly assigned rodents<br>2) Not equally treated rodents except for the intervention
Outcome measure(s) (O)	Inclusion criteria: 1) Studies that reported cognitive improvement or deterioration as their outcomes.<br>2) Studies that reported differences in compositionof gut microbiota as their outcomes.<br>3) Studies that reported differences in biomarkers, or expression of protein or mRNA as their outcomes.<br>Exclusion criteria: Studies without any of inclusion outcomes described above.

Study selection and data extraction:	
Procedure for study selection and<br>Prioritise the exclusion criteria	Two autors (S.S and S.K) will individually screen all the titles and abstracts of publications<br>obtained by database search using PubMed, EMBASE, Cochrane and Web of Science.<br>Then we will exclude less releavant articles according to inclusion criteria described<br>in sections of P, I, C, O above. Any disagreements will be resolved by consulting ▯ ▯ ▯<br>Additional citations will be sought using reference lists and tables of relevant articles,<br>and gray literature. We will exclude all the publications not written in English or not available full text online
Study designs to be included	Inclusion criteria: Randomized, double-blind, placebo-controlled study<br>Exclusion criteria: Not randomized, double-blind, placebo-controlled study
Primary outcome(s)	Cognitive improvement or deterioration assessed by behavioral tests for investigate learning and<br>memory function of rodents including Morris water maze test, Novel objective recognition test<br>or open field test etc.
Secondary outcome(s)	Changes in biomarkers such as inflammatory markers including IL-1beta, IL-6, IL-10 in blood.<br>Changes in expression of protein and/ or mRNA that is thought to be important for keeping<br>the normal brain function including BDNF, 5-HT and bdnf etc.
Risk of bias and quality assessment	Two authors (S.S., S.K.) independently assessed the quality of studies using SYRCLE's risk of bias tool<br>to assess the quality of animal studies, which includes 1) selection bias, 2) performance bias,<br>3) detection bias, 4) attrition bias and 5) reporting bias. Any disagreement will be resolved<br>by consulting○○○
