# Supplementary material for: The effects of microbiome-targeted therapy on cognitive impairment and postoperative cognitive dysfunction—A systematic review
Source: PLoS One. 2023 Feb 7;18(2):e0281049. doi: 10.1371/journal.pone.0281049 (PMC9904456; doi:10.1371/journal.pone.0281049)
Supplement: S4 File — (PDF) [file pone.0281049.s004.pdf]

First Author, Year, DOI
Aisu, 2015, 10.3892/etm.2015.2640
Anderson, 2004, 10.1136/gut.2003.024620
Capurso, 2019, 10.1097/MCG.0000000000001170
Chen, 2016, 10.1016/j.soard.2015.07.010
Consoli, 2016, 10.1177/0148607115584387
Darbanidi, 2020, 10.1016/j.clnu.2019.11.008
De Andrade, 2017, 10.21037/tgh.2017.08.01
Forestier, 2008, 10.1186/cc6907
Guillemard, 2010, 10.1017/S0007114509991395
Hempel, 2012, 10.1001/jama.2012.3507
Horvat, 2010, 10.1007/s00508-010-1347-8
Kanazawa, 2005, 10.1007/s00423-004-0536-1
Komatsu, 2016, 10.1007/s00595-015-1178-3
Kotzampassi, 2015, 10.1007/s00268-015-3071-z
Liu, 2011, 10.1111/j.1365-2036.2010.04492.x
Liu, 2013, 10.3945/ajcn.112.040949
Makino, 2010, 10.1017/S000711451000173X
McNaught, 2002, 10.1136/gut.51.6.827
McNught, 2002, 10.1136/gut.51.6.827
Mizuta, 2016, 10.12938/bmfh.2015-017
Morrow, 2010, 10.1164/rccm.200912-1853OC
Nazari, 2020, 10.30491/TM.2021.250469.1176
Ohigashi, 2011, 10.1007/s00595-010-4450-6
Okazaki, 2013, 10.1016/j.nut.2013.03.015
Park, 2020, 10.3988/jcn.2020.16.2.292
Pellino, 2013, 10.1186/1471-2482-13-s2-s57
Qu, 2019, 10.1371/journal.pone.0211233
Rayes, 2002, 10.1055/s-2002-35259
Serban, 2014, 10.1016/j.canlet.2013.08.013
Shimizu, 2009, 10.1007/s10620-008-0460-2
Shimizu, 2018, 10.1186/s13054-018-2167-x
Sommacal, 2015, 10.1080/01635581.2015.1004734
Stadlbauer, 2019, 10.3920/bm2018.0067
Tanaka, 2012, 10.1016/j.surg.2012.02.021
Wei, 2016, 10.1186/s13054-016-1491-2
Yamada, 2015, 10.1177/0148607114529596
Zhang, 2012, 10.1097/MAJ.0b013e31823aace6

First Author, Year, DOI
Alam, 2018, 10.1016/j.ebiom.2018.10.021

Bischoff, 2016, 10.1097/MCO.0000000000000242
Calder, 2017, 10.1016/j.arr.2017.09.001
Camfield, 2011, 10.1017/s0007114511000158
Caracciolo, 2014, 10.1016/j.mad.2013.11.011
Cascella, 2018, 10.23736/s0375-9393.17.12146-2
Corpino, 2017, 10.7363/060205
Coutts, 2020, 10.1007/s41999-020-00396-x
Cryan, 2012, 10.1038/nrn3346
Dalile, 2019, 10.1038/s41575-019-0157-3
Eastwood, 2021, 10.1016/j.neubiorev.2021.06.032
Ellison, 2016, 10.1017/s0007114516003354
Haak, 2017, 10.1097/mcc.0000000000000389
Hopkins, 2001, 10.1136/gut.48.2.198
Kasatpibal, 2017, 10.1093/cid/cix114
Li, 2018, 10.1016/j.ejphar.2018.05.003
Luo, 2019, 10.1007/s10787-018-00559-0
Malaguarnera, 2012, 10.1007/s12603-011-0357-1
Manzanares, 2016, 10.1186/s13054-016-1434-y
McDonald, 2016, 10.1128/mSphere.00199-16
McLoughlin, 2017, 10.3945/ajcn.117.156265
Mello, 2016, 10.1159/000443350
Novotny, 2019, 10.3389/fnagi.2019.00170
Romo-Araiza, 2020, 10.1016/j.mehy.2019.109410
Ruiz-Gonzalez , 2021, 10.1016/j.pnpbp.2020.110189
Schlegel, 2019, 10.3233/JAD-190460
Scott, 2017, 10.1016/j.bbi.2017.02.004
Serra, 2019, 10.1007/s13668-019-0265-2
Skonieczna-Zydecka, 2018, 10.3390/jcm7120556
Skvarc, 2018, 10.1016/j.neubiorev.2017.11.011
Subramaniyan, 2019, 10.1213/ane.0000000000004053
Ticinesi, 2018, 10.2147/cia.S139163
Ticinesi, 2019, 10.1007/978-3-030-25650-0_8
Xu, 2020, 10.18632/aging.103738
Yi, 2016, 10.1016/j.ajic.2015.12.001
Zhan, 2018, 10.18632/aging.101464
Zhang, 2019, 10.1111/cns.13103

First Author, Year, DOI

de Vrese, 2006, 10.1016/j.vaccine.2006.05.048
Manzoni, 2017, 10.1016/j.foodres.2017.06.005
Kim, 2020, 10.1093/gerona/glaa090

Nagpal, 2019, 10.1016/j.ebiom.2019.08.032
Neto, 2013, 10.3390/nu5041276
Oh, 2020, 10.3390/nu12030837
Provasi, 2016, 10.1016/j.neurobiolaging.2016.08.019
Scholey, 2013, 10.1186/1745-6215-14-404
Szczechowiak, 2019, 10.1016/j.pbb.2019.172743
Wei, 2015, 10.1186/s12879-015-0973-1
Winkler, 2005, 10.5414/CPP43318

Allen, 2013, 10.3310/hta17570
Mego, 2015, 10.1016/j.ctim.2015.03.008
Orenstein, 2018, 10.1093/cid/cix736
Van Beurden, 2017, 10.1177/1756283X17690480

Asemi, 2016, 10.1016/j.clnu.2015.07.009
de Groot, 2017, 10.1371/journal.pone.0188475
Gonai, 2017, 10.3920/bm2016.0230
Kellow, 2014, 10.1186/1472-6823-14-55
Parekh, 2016, 10.3389/fendo.2016.00055
Majewska, 2020, 10.3390/jcm9040998
Kobyliak, 2020, 10.1016/j.obmed.2020.100248

First Author, Year, DOI
Aqaeinezhad, 2018, 10.1007/s10787-017-0436-y
Amirani, 2020, 10.15171/mejdd.2020.160
Blanchet-Réthoré, 2017, 10.2147/CCID.S135529
Eguchi, 2011, 10.1016/j.amjsurg.2010.02.013
González-Hernández, 2012, 10.1186/1475-2891-11-90
Grat, 2017, 10.1016/j.clnu.2017.04.021
Haidmayer, 2020, 10.3390/nu12082337
Karimi, 2005, 10.1358/dot.2005.41.7.917341
Kruis, 2004, 10.1136/gut.2003.037747
Palumbo, 2016, 10.5507/bp.2016.044
Pianta, 2017, 10.1002/art.40003
Shinkai, 2013, 10.1017/S0007114512003753
Singh, 2013, 10.1038/ejcn.2012.197
Steed, 2010, 10.1111/j.1365-2036.2010.04417.x
Stofilova, 2017, 10.1016/j.biopha.2017.07.138
Tsuda, 2007, 10.1080/00365520701396091
Vaughn, 2016, 10.1097/mib.0000000000000893
Watts, 2016, 10.1089/acm.2016.0115

Wildt, 2006, 10.1097/01.MIB.0000218763.99334.49
Wildt, 2011, 10.1016/j.crohns.2010.11.004
Yamamoto, 2016, 10.3168/jds.2015-10605
Zamani, 2016, 10.1111/1756-185X.12888

Huang, 2016, 10.3390/nu8080483
Mohammadi, 2016, 10.1179/1476830515y.0000000023

Reasons for Exclusion
Perioperative studies, but focused on other than cognitive function such as the occarences of SSI and VAP, or the level of inflammatory cytokines.

Reasons for Exclusion
Reviews or Not interventional studies

Reasons for Exclusion
Populations are not of interest

11

Studies on antibiotics-associated diarrhea or diarrhea caused by C.difficile

4

Studies on Diabetes, Metabolic syndorome

7

Reasons for Exclusion

Studies on Immunological disease including Rheumatoid arthritis,
Atopic dermatitis, Allergic, Ollagenous colitis, Crohn's disease, Ulcerative colitis
