# Supplementary figures and images for: The effects of microbiome-targeted therapy on cognitive impairment and postoperative cognitive dysfunction—A systematic review
Source: PLoS One. 2023 Feb 7;18(2):e0281049. doi: 10.1371/journal.pone.0281049 (PMC9904456; doi:10.1371/journal.pone.0281049)

## Slide 1
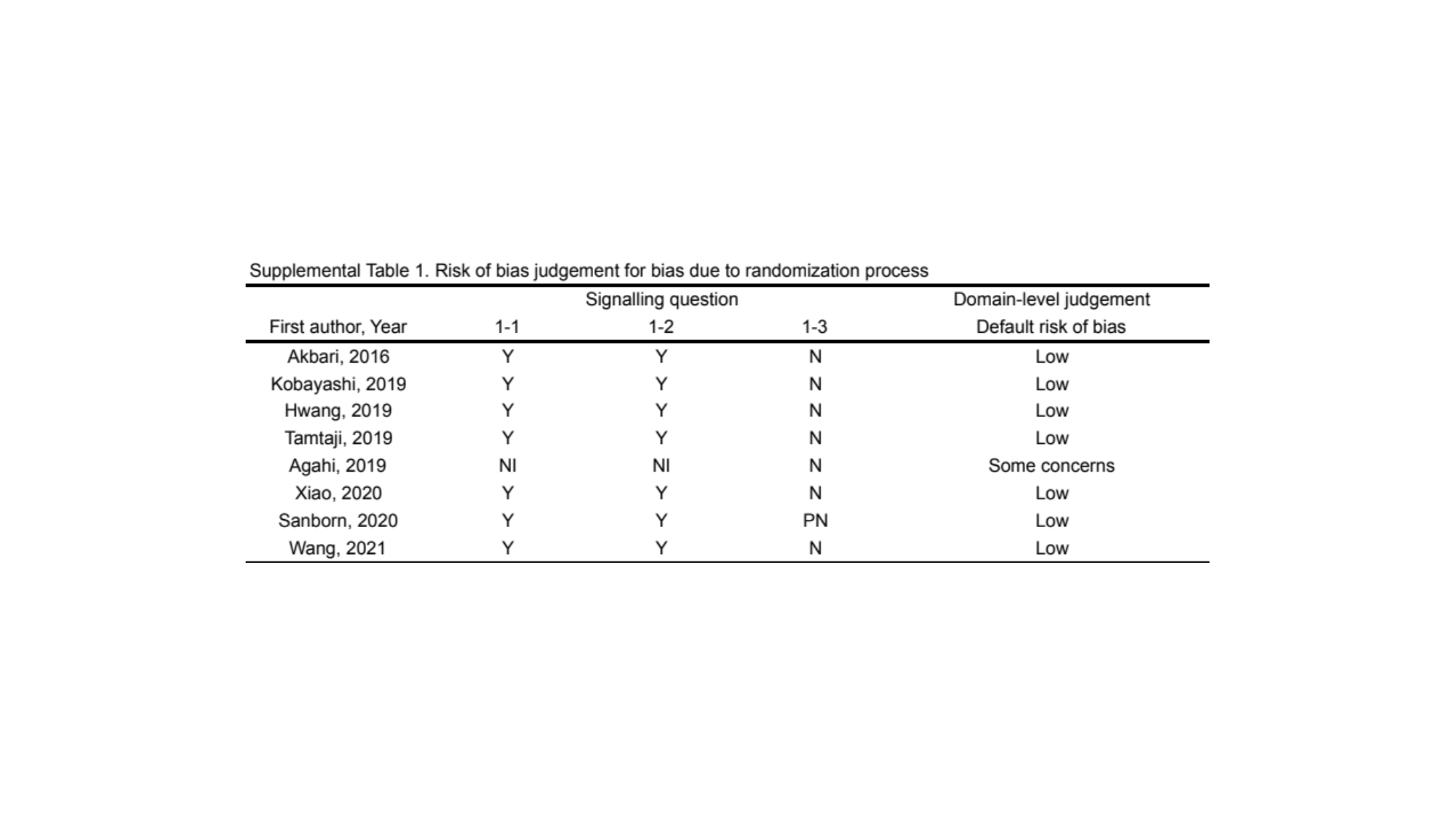

## Slide 2
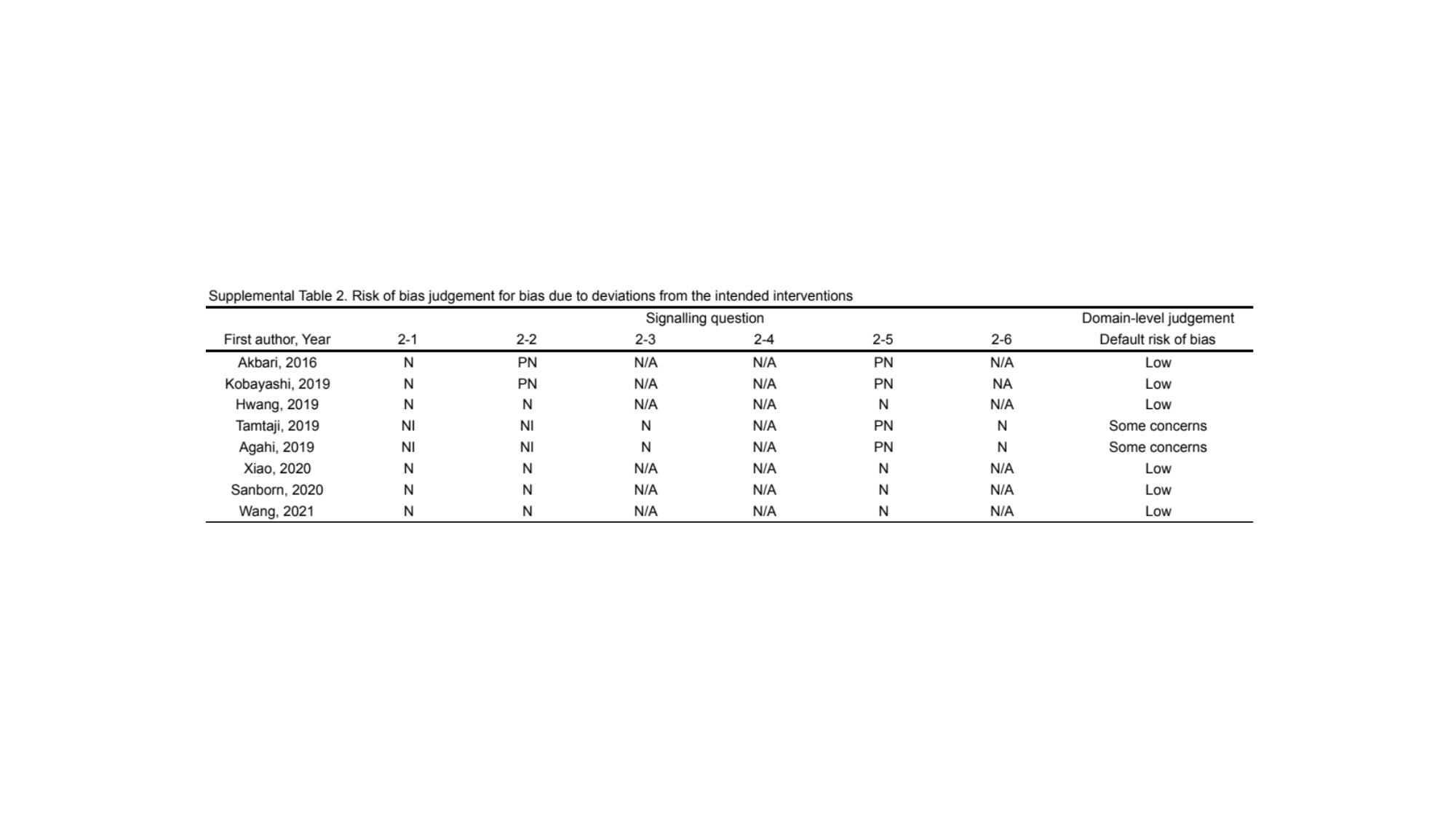

## Slide 3
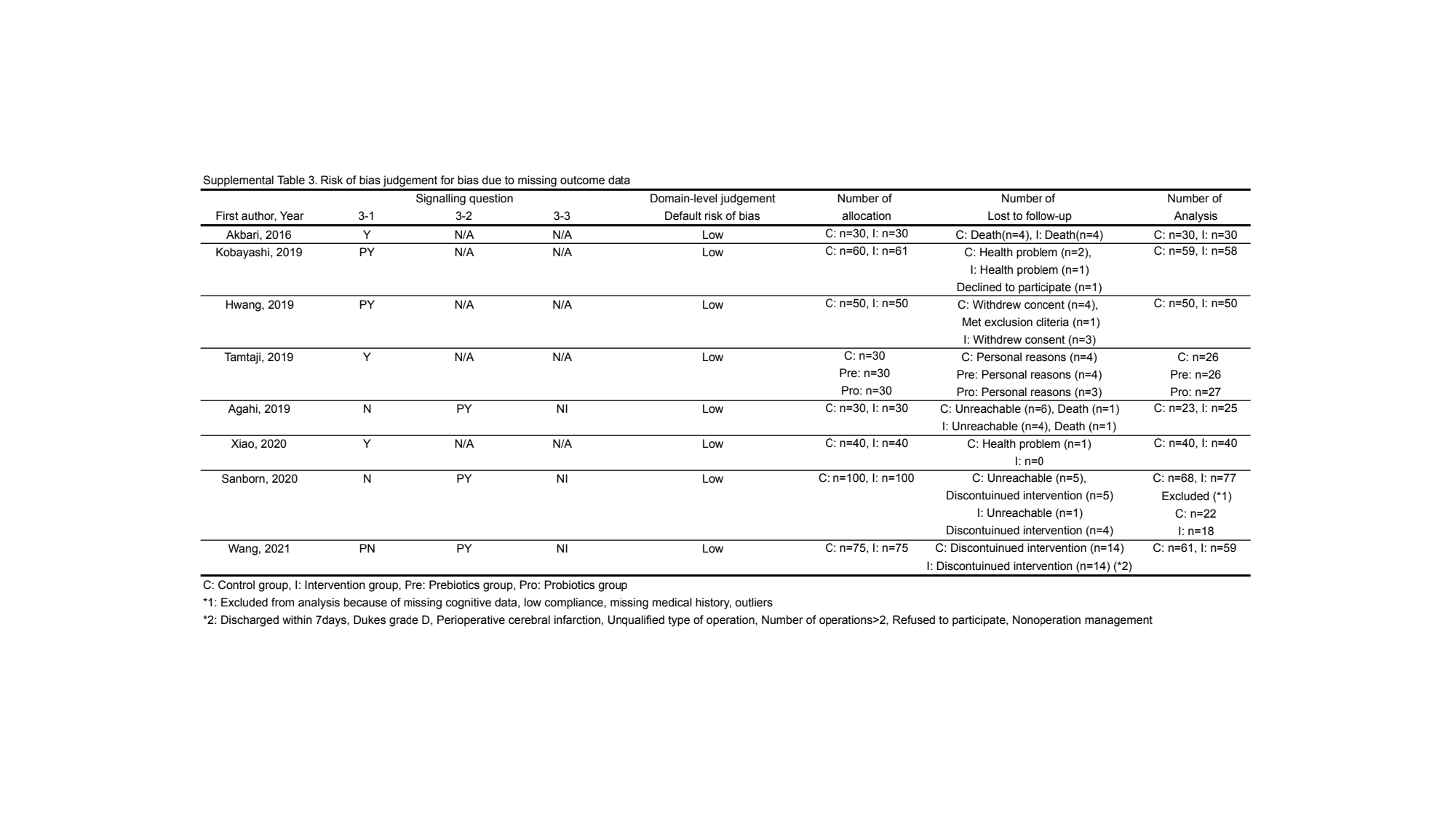

## Slide 4
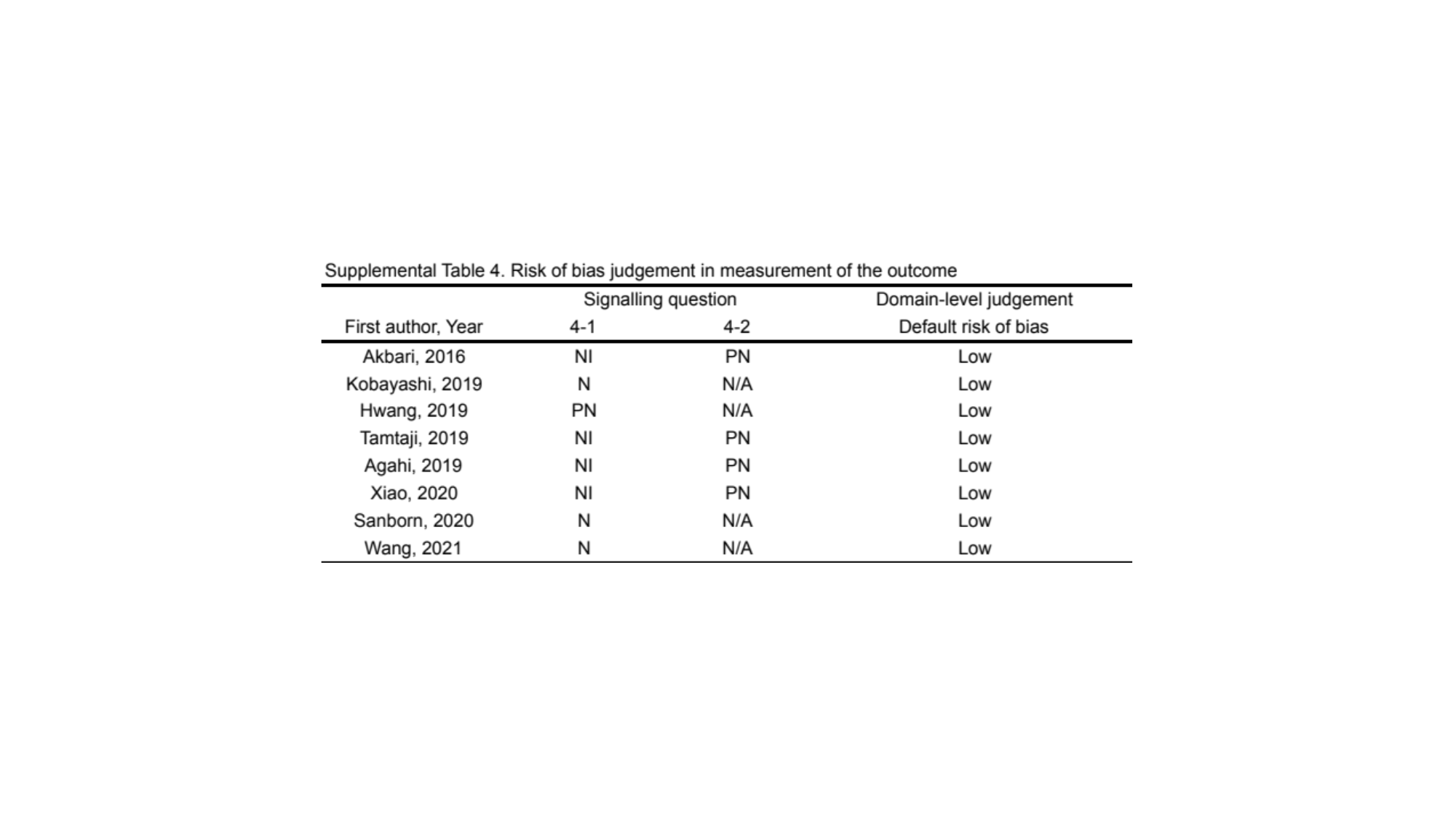

## Slide 5
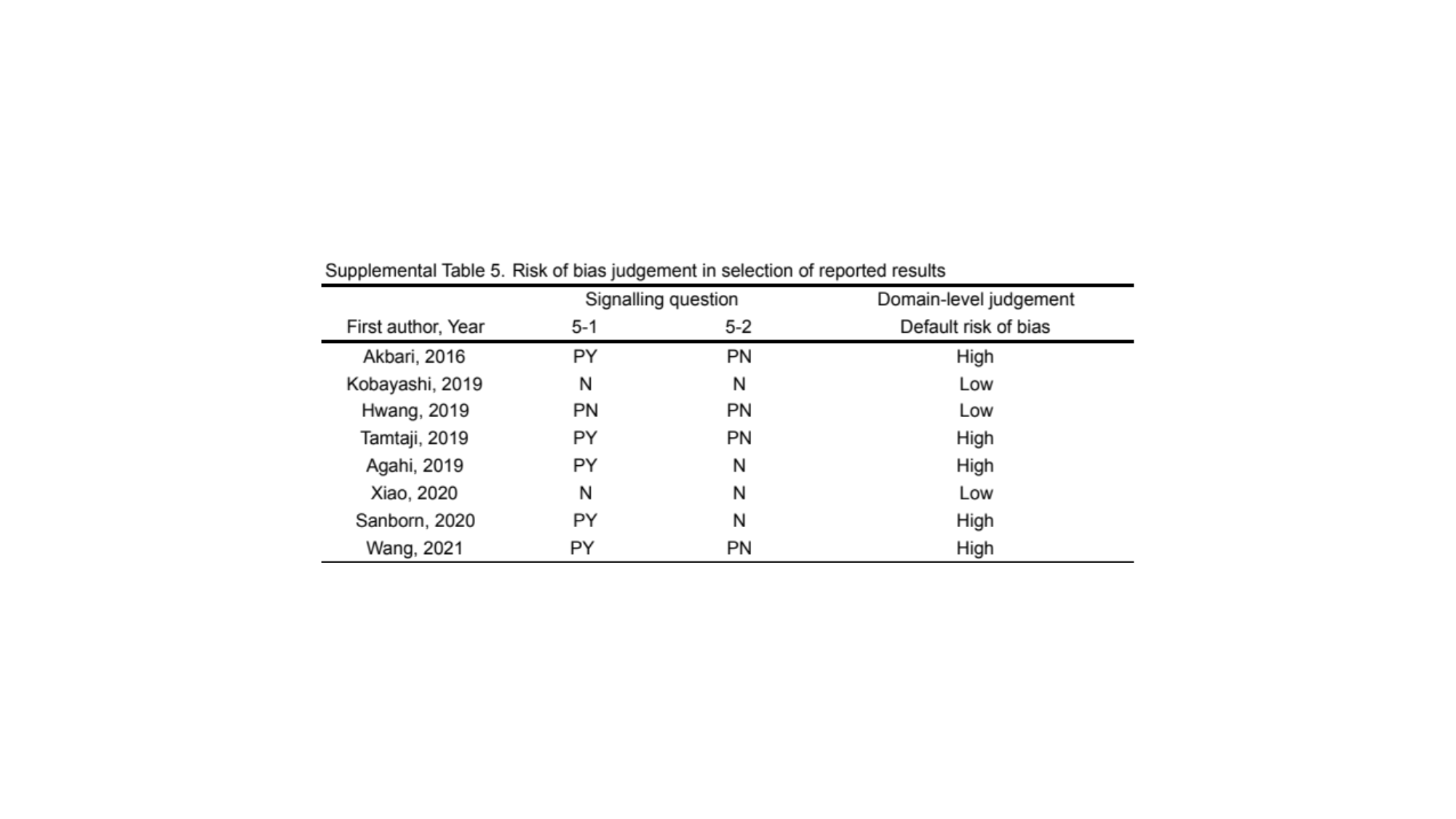

## Slide 6
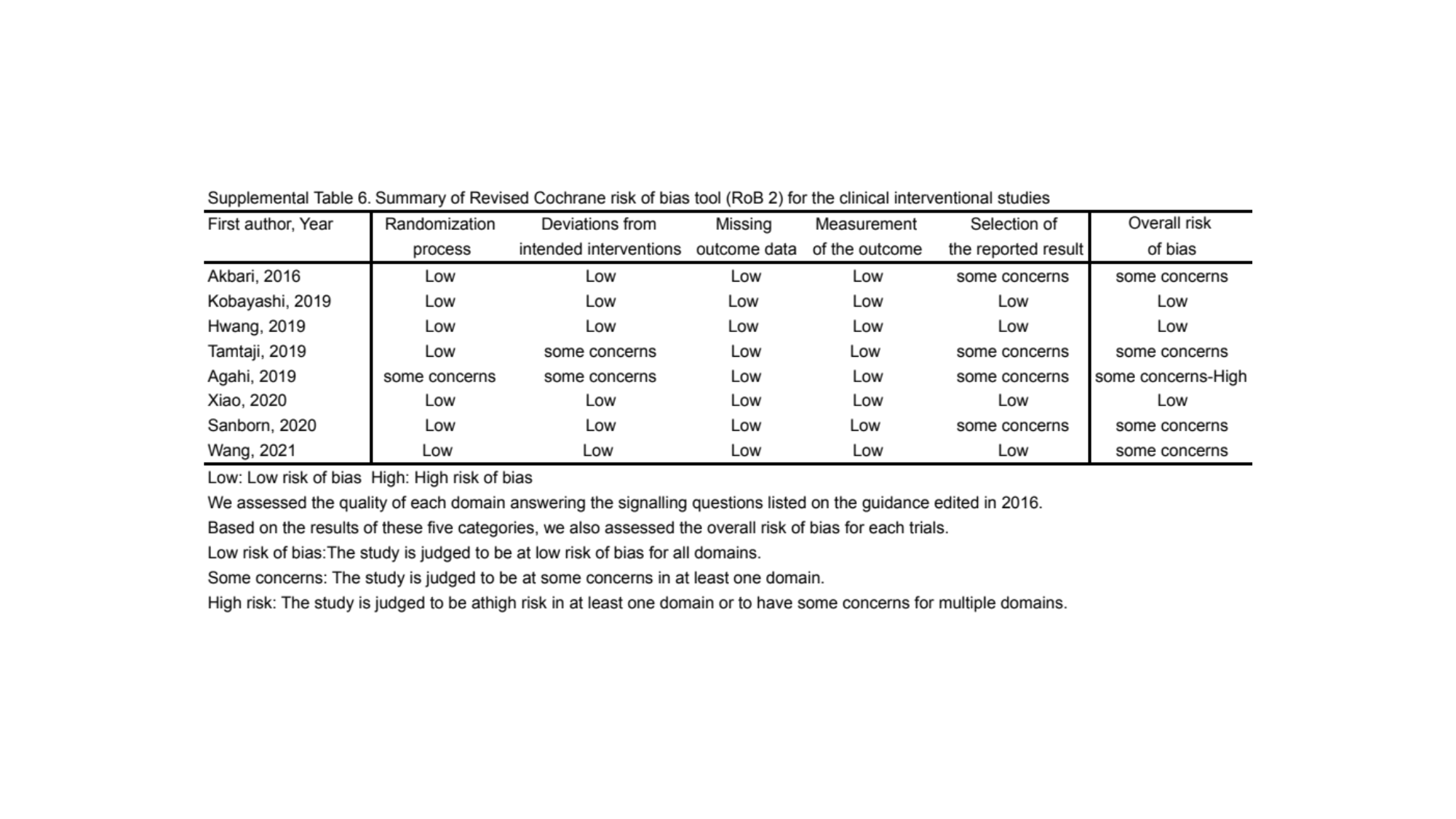

## Slide 7
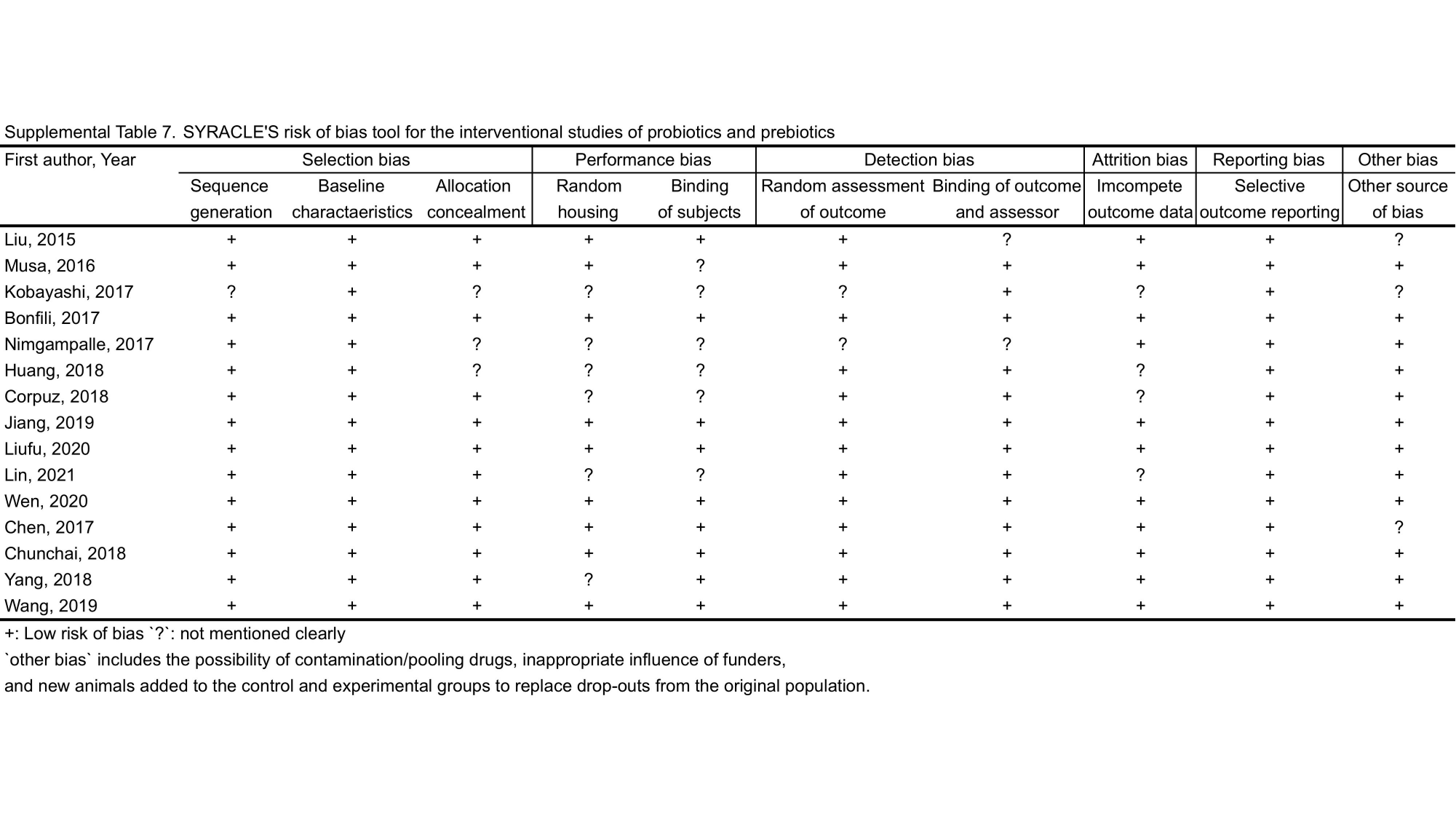

Supplement: S5 File — (PPTX) [file pone.0281049.s005.pptx]
