# Supplementary material for: The effects of microbiome-targeted therapy on cognitive impairment and postoperative cognitive dysfunction—A systematic review
Source: PLoS One. 2023 Feb 7;18(2):e0281049. doi: 10.1371/journal.pone.0281049 (PMC9904456; doi:10.1371/journal.pone.0281049)
Supplement: Attachment — Submitted filename: Editor comments-PLOS one.docx [file pone.0281049.s006.docx]

Thank you for your comments. Please see our responses below.

In the case of your submission, we are concerned that the date range over which the literature review was conducted have not been adequately reported. Please note that systematic and scoping reviews should provide an up-to-date overview of the research topic covered. Since it is not clear whether the literature search was completed over a year ago, we are concerned that this study does not meet this requirement.

**We have redone a comprehensive literature search, added newer articles, revised the manuscript and updated supporting documents.**

Furthermore, should you choose to resubmit please also include a copy of the full electronic search strategy used for at least one database as supporting file.

**A table of search strategy was added in supporting information.**
