# Supplementary material for: The effects of microbiome-targeted therapy on cognitive impairment and postoperative cognitive dysfunction—A systematic review
Source: PLoS One. 2023 Feb 7;18(2):e0281049. doi: 10.1371/journal.pone.0281049 (PMC9904456; doi:10.1371/journal.pone.0281049)
Supplement: Attachment — Submitted filename: PLOS one reviewer comments 4-30-22-Final.docx [file pone.0281049.s007.docx]

Thank you for your constructive comments and suggestions. We have revised the manuscript accordingly. Please see our responses in red.

The main changes were:

1. The change in the risk of bias assessment tool. We used the updated version, which provides more detailed and accurate assessment. Please see our supplemental materials.
2. We revised the Discussion extensively and added Gap in the Knowledge.
3. Added a Table of Commercially Available Products.
4. Study protocol and exclusion criteria were added as Supplemental Materials.

PONE-D-21-36898

The Effects of Microbiome-Targeted Therapy on Cognitive Impairment and Postoperative Cognitive Dysfunction - A Systematic Review

PLOS ONE


Journal Requirements:

When submitting your revision, we need you to address these additional requirements.

1. Please ensure that your manuscript meets PLOS ONE's style requirements, including those for file naming. The PLOS ONE style templates can be found at

[https://journals.plos.org/plosone/s/file?id=wjVg/PLOSOne_formatting_sample_main_body.pdf](https://urldefense.com/v3/__https:/journals.plos.org/plosone/s/file?id=wjVg*PLOSOne_formatting_sample_main_body.pdf__;Lw!!LQC6Cpwp!_djqaxBCc5_YmqH_zCfegv9pGmnQqoa9u7XJfkKzX6J41mwHMLcy-3dJhQjU7h2uufg$) and

[https://journals.plos.org/plosone/s/file?id=ba62/PLOSOne_formatting_sample_title_authors_affiliations.pdf](https://urldefense.com/v3/__https:/journals.plos.org/plosone/s/file?id=ba62*PLOSOne_formatting_sample_title_authors_affiliations.pdf__;Lw!!LQC6Cpwp!_djqaxBCc5_YmqH_zCfegv9pGmnQqoa9u7XJfkKzX6J41mwHMLcy-3dJhQjUpNJbmmw$)

We have formatted the manuscript according the journal’s guideline.

2. In your Data Availability statement, you have not specified where the minimal data set underlying the results described in your manuscript can be found. PLOS defines a study's minimal data set as the underlying data used to reach the conclusions drawn in the manuscript and any additional data required to replicate the reported study findings in their entirety. All PLOS journals require that the minimal data set be made fully available. For more information about our data policy, please see [http://journals.plos.org/plosone/s/data-availability](https://urldefense.com/v3/__http:/journals.plos.org/plosone/s/data-availability__;!!LQC6Cpwp!_djqaxBCc5_YmqH_zCfegv9pGmnQqoa9u7XJfkKzX6J41mwHMLcy-3dJhQjUZVQ_z-k$).

Upon re-submitting your revised manuscript, please upload your study’s minimal underlying data set as either Supporting Information files or to a stable, public repository and include the relevant URLs, DOIs, or accession numbers within your revised cover letter. For a list of acceptable repositories, please see [http://journals.plos.org/plosone/s/data-availability#loc-recommended-repositories](https://urldefense.com/v3/__http:/journals.plos.org/plosone/s/data-availability*loc-recommended-repositories__;Iw!!LQC6Cpwp!_djqaxBCc5_YmqH_zCfegv9pGmnQqoa9u7XJfkKzX6J41mwHMLcy-3dJhQjUhqDd_xI$). Any potentially identifying patient information must be fully anonymized.

Important: If there are ethical or legal restrictions to sharing your data publicly, please explain these restrictions in detail. Please see our guidelines for more information on what we consider unacceptable restrictions to publicly sharing data: [http://journals.plos.org/plosone/s/data-availability#loc-unacceptable-data-access-restrictions](https://urldefense.com/v3/__http:/journals.plos.org/plosone/s/data-availability*loc-unacceptable-data-access-restrictions__;Iw!!LQC6Cpwp!_djqaxBCc5_YmqH_zCfegv9pGmnQqoa9u7XJfkKzX6J41mwHMLcy-3dJhQjUJrg3wCA$). Note that it is not acceptable for the authors to be the sole named individuals responsible for ensuring data access.

We will update your Data Availability statement to reflect the information you provide in your cover letter.

Minimal data set are available in the supporting information files.

Editor Comments:

1- Methods. It is unclear if the study followed a protocol and if the methodology was conceived before study start. Did you register the protocol before study start? The lack of a registered protocol is a major limitation that should be discussed.

We have added more information the protocol we used (Eligibility Criteria) in the Methods.

Since this systematic review includes clinical and animal studies, we attempted to register clinical studies and animal studies separately. However, PROSPERO changed registration requirements in October 2019 in the midst of our literature search and no longer allowed registration once a literature search started. (<https://www.crd.york.ac.uk/prospero/#aboutregpage>). Therefore, we did not seek approval at that time. However, during the COVID-19 pandemic, they made exceptions on the registration process and our clinical study was approved due to COVID-19 pandemic (ID: CRD42020178197). The link is

<https://www.crd.york.ac.uk/prospero/display_record.php?RecordID=178197>. (PDF is attached as Supplemental).

We did not know of this approval until recently. An application for the animal study was not sought out.

2- Methods. The study included “aged” patients. Did you follow any cut-off? Any age limit?

There was no age cut-off or age limit, patients’ ages are between 50-100 years old. This information was added to the ‘Results’ section.

3- Methods. How did you manage missing outcome data? Did you contact corresponding authors?

Missing data were due to loss of follow-up or death in these studies. We created a table of signaling questions on the risk of bias assessment, which was added as a supplemental.

4- Methods. Supplemental details on risk of bias assessment should be reported in the supplementary material (e.g.: what other bias include? How did you finally rate a study? etc.)

The domain of other risk of bias in STRACLE’s risk of bias tool includes: The possibility of contamination or pooling of drugs; added animals to control or intervention groups to replace drop-outs from the original population; and inappropriate influence of funders.

For each clinical trial, we assessed its quality using the overall risk of bias. Details are available in Supplemental Table 2.

5- Results. References of major exclusions should be included in the supplementary material.

We excluded articles investigating the effects of MTT on other than cognitive function. We included this information in the supplementary material.

6- Results. Much more details on risk of bias assessment should be reported in the supplementary material (full details on each item). The authors reported that all items of each study had low risk of bias; this is possible but very improbable.

We changed the assessment method from the original Cochrane risk of bias tool to the Revised Cochran risk of bias tool (RoB2) in this manuscript which is a more detailed risk of bias assessment tool. Based on the RoB2 protocol, we assessed the quality of each domain by answering the signaling questions. They were uploaded as supporting files.

7- Results. Overall risk of bias evaluations for each trial should be reported.

Briefly, we assigned “low risk of bias” when a study is judged to be low risk of bias for all domains. Accordingly, “some concerns” was when a study is judged to be “some concerns” at least one domain.; “High risk of bias” was when a study is judged to be high risk of bias at least one domain or have “some concerns” for multiple domains.

8- Discussion. The certainty of evidence supporting microbiome-target therapy is very low. This therapy that should be tested in large, randomized trials.

Authors agree with above statement and included this in the ‘Discussion’.

9- Discussion. A paragraph on study’s limitations should be included.

We have added several limitations.

Minor comments:
1- Methods. I suggest to include a PRISMA 2020 checklist.

Done.

2- Results. A PRISMA 2020 flow-chart should be used.

Done.


Reviewers' comments

Reviewer #1:

1 As the authors maintained, neuroinflammation may be an important factor of PND. The question is whether neuroinflammation is caused by abnormal gut microbiota, or surgery-induced peripheral inflammation?

This is a good point, and we added a discussion of the potential contribution of surgery induced inflammation on neuroinflammation.

2 We know that FMT will not see significant changes in the short term. At least, the regulation of brain function by FMT is a chronic process. Is it better to treat before or after surgery?

It is still not clear how the timing of MMT including FMT affects cognitive outcomes. It is one of the important areas requiring further investigation. We added a paragraph regarding this gap of knowledge, and recommendations for further research in the ‘Discussion’.

Reviewer #2:

Methods: should be more specific in the abstract.

We have updated the Methods in Abstract.

Was this systematic review registered?

Please see our response to the editor’s comment No. 1.

Readers will benefit from separate Exposure and outcomes section in the method. which probiotics/ how they are usually administered etc. A table showing risk/benefit/available commercial product [for human or experimental use], of MMT will be useful. This will highlight differences between various interventions.

We have added a new table on commercially available products.

Discussion: While the authors summarized the current research findings. They should discuss, based on the research, which specific MMT regimen shows promise and should be investigated further. The goal is to guide readers and researcher, what next? How these MMT should be studied?

Authors did write “further basic and clinical research, and more randomized controlled studies are needed that correlate specific probiotic and prebiotic formulas with measurable biological and clinical outcomes relevant to cognitive impairment” Some specifics should be added rather than a generalized statement.

The ‘Discussion’ section has been revised extensively and now includes a section on recommendations for further research.

Assessment of outcome, POCD is complicated by varied resource intensive methods, and should be discuss further.

We added a sentence about neurocognitive tests in the Discussion.

Also, how much improvement in POCD can result from MMT should be discussed. Given perioperative POCD pathophysiology is not fully understood.

We added more information about the single human RCT in the perioperative setting (Wang et. al, 2021) on the Results section.

“Please correct word spacing in tables”. The main results seem to be copy pasted. Please be consistent and specific, tell what improved and by how much (if possible) and was it significant?

We revised the tables by adding more information in the results.

Reviewer #3:

Abstract:
1) The conclusion does not really convey much information other than a positive outlook on the use of microbiome interventions. The authors should either shorten it, or better yet offer some more sophisticated interpretation of their results.

We added a sentence in the ‘Results’ section of the Abstract; “most showed that MTTs decrease inflammation by down-regulating inflammatory cytokines and oxidative stress in both perioperative and non-perioperative settings”.

Introduction:
1) Given that many of the studies reviewed here are conducted in animals, the authors should discuss the similarities and differences in human and animal model microbiota and they should give a clear understanding of the limitations and benefits of studies in each.

We included the information on the differences in species of gut microbiota between human and animals.

2) The authors should include a paragraph on what has been published in human and animal models about the changes in the microbiota that are seen in the perioperative setting due to surgery, anesthesia, oxygen, opiates, and other known causes of dysbiosis. It is important to set up for the reader why it is likely that disruption of the microbiota are a cause of POCD.

Thank you for the suggestion. We have added more information on the causes of POCD in the “Introduction”.

Methods
1) A reference should be given for the Cochrane risk and bias tool

The reference has been added.

2) It would be helpful and provide useful validation for this work if the authors cited other published studies that use similar literature review methodologies.

The paper below assessed 3 RCTs on the effect of probiotics for dementia using the older version of Risk of bias assessment. Our revised manuscript used an updated version of risk of bias assessment (Rob2), therefore, we believe that our risk of bias assessment is more thorough.

Jenifer F Krüger, Elaine Hillesheim, Amanda C S N Pereira, Carolina Q Camargo, Estela I Rabito, Probiotics for dementia: a systematic review and meta-analysis of randomized controlled trials, Nutrition Reviews, Volume 79, Issue 2, February 2021, Pages 160–170.

Results
1) It would be helpful if the authors came up with a diagram that summarized the various mechanisms by which dysbiosis is thought to alter cognitive outcomes that also includes some indication of how the interventions might have helped.

Please see Figure 2 which illustrates the possible mechanisms of the effect of microbiome on cognition.

Discussion
1) It would be great if the authors could include a paragraph suggesting where the gaps are in the literature and what should be done next to address them. This is a really helpful feature of many reviews of this kind.

The ‘Discussion’ section has been revised extensively and now includes a section on recommendations for further research.
