# Supplementary material for: The effects of microbiome-targeted therapy on cognitive impairment and postoperative cognitive dysfunction—A systematic review
Source: PLoS One. 2023 Feb 7;18(2):e0281049. doi: 10.1371/journal.pone.0281049 (PMC9904456; doi:10.1371/journal.pone.0281049)
Supplement: Attachment — Submitted filename: PLOS -reviewer comments 7-7-SK.docx [file pone.0281049.s008.docx]

Thank you for your comments and suggestions. Please see our responses in red.

We also corrected typos and grammar errors in the manuscript.

Editor Comments:


1-Abstract. I suggest to report that you included animal and human studies.

Done.

2-Results. SYRCLE risk of bias results should be reported. Full details on each study should be reported in the supplementary material.

The results of the SYRCLE’s risk of bias were added. A table of SYRCLE’s risk of bias was added as Table 7 in Supplementary Material (S7).

3-Results. The structure of the Results section is unclear and confusing in my opinion. Results should include systematic review results, an objective assessment of the evidence. Statements on potential effects or interpretation of the results shuld be moved to the discussion section.
I suggest to report results of systematic review in the Results section (“Gut-microbiota and MTTs on Cognitive Function in Rodents” and “Gut-microbiota and MTTs on Cognitive Function in Humans”).
In my opinion the paragraph “Neuroinflammation, aging, and POCD” and “Microbiome-targeted therapy (MTT)” may be moved in the discussion.

As suggested, these paragraphs have been moved to the Discussion. Discussion was restructured to read better.

4-Supplementary material. No need to include the PROSPERO protocol in the supplementary material; it is online and freely available. Please remove it.

Done.

5-Supplementary material. The PRISMA 2020 checklist for the abstract is missing.

It has been added.

6-Supplementary material. The ‘Microbiome Exclusion-Final’ table should include information on major exclusions. Some more information allowing the retrieval of each manuscript should be reported (e.g.: first author + year of publication + doi; first author + year of publication + journal info; full reference according to journal style). The actual form (first author + year of publication) is not informative enough (the manuscripts could not be retrieved).

Done. (first author, year, doi)

6. Review Comments to the Author


1. The introduction is considerably undercited. There is a wealth of literature on the gut and other microbiota and surgical outcomes that should be cited in a more complete fashion. Also, the authors note that anesthetics, analgesics, and antibiotics have effects on the microbiota but do not have any citations to back this assertion.

More information and citations were added to the Introduction.

2. There are several other reviews of probiotics and surgical outcomes. It’s important to frame the introduction to distinguish how this manuscript is a valuable addition to the literature.

We have added a sentence in the Introduction.

3. While systematic reviews of this kind are less common in animal models than in clinical studies and the practices for them are less clear, they should still attempt to conform to the better recognized approach for clinical studies. I would argue that the preclinical approach should also have been registered/deposited as well.

As editor suggested, the SYRCLE’s risk of bias was added. Unfortunately, we were not able to register the part of our project (animal studies) due to the policy change in PROSPERO. Please see below (our previous response regarding study registration)

……………………………………………………………………………………………………………………………………………………..

PROSPERO changed registration requirements in October 2019 in the midst of our literature search and no longer allowed registration once a literature search started. (<https://www.crd.york.ac.uk/prospero/#aboutregpage>). Therefore, we did not seek approval at that time. However, during the COVID-19 pandemic, they made exceptions on the registration process and our clinical study was approved due to COVID-19 pandemic (ID: CRD42020178197).

r
4. What is gray literature?

We considered gray literature as proceedings or theses.

5. Can the authors provide some citations validating their methodology for assessing bias? Particularly as it relates to animal studies.

The reference below was added in the Methods section.

Ma, LL et al. Methodological quality (risk of bias) assessment tools for primary and secondary medical studies: what are they and which is better? Mil Med Res. 2020 Feb 29;7(1):7. doi: 10.1186/s40779-020-00238-8

6. The vast majority of studies were excluded due to lack of relevance. This does suggest the possibility that the search terms were not well designed for the study.

This was added to the Limitations.
